# Video indirect ophthalmoscopy using a hand-held video camera

**DOI:** 10.4103/0301-4738.73718

**Published:** 2011

**Authors:** Mahesh P Shanmugam

**Affiliations:** Sankara Eye Hospital, Airport–Varthur Road, Kundalahalli Gate, Bangalore - 560 038, Karnataka, India

**Keywords:** Fundus imaging, hand-held video camera, video indirect ophthalmoscopy

## Abstract

Fundus photography in adults and cooperative children is possible with a fundus camera or by using a slit lamp-mounted digital camera. Retcam™or a video indirect ophthalmoscope is necessary for fundus imaging in infants and young children under anesthesia. Herein, a technique of converting and using a digital video camera into a video indirect ophthalmoscope for fundus imaging is described. This device will allow anyone with a hand-held video camera to obtain fundus images. Limitations of this technique involve a learning curve and inability to perform scleral depression.

Photography of fundus pathology is often necessary for documentation, diagnostic and medicolegal purposes. Serial fundus photography helps assess response to treatment. Photographic documentation of fundus lesions is particularly essential in infants and children with diseases such as retinoblastoma.

It is possible to obtain good fundus images for documentation in cooperative adults using the fundus camera as well as the slit lamp-mounted digital camera, although with some difficulty.[1] It may be possible to use these instruments to image the fundus of cooperative, awake children. It is however extremely difficult to obtain fundus images of infants with a routine fundus camera even under anesthesia.

Techniques such as custom mounting the fundus camera vertically, hand-held fundus camera, video indirect ophthalmoscope and the Retcam™ have been employed to image the fundii of children under general anesthesia.[2] Each technique has its own limitation apart from being expensive. Mounting a fundus camera vertically would mean sacrificing a fundus camera for a need that occurs rarely. The RetCam™is prohibitively expensive, the hand-held fundus camera and the video indirect ophthalmoscope being the less-expensive choices. Importantly, most general ophthalmologists would be averse to purchasing fundus photography equipment that they would use rarely and the capital needed for purchase of these equipment may ideally be used to acquire other ophthalmic equipments that are used more often. Hand-held digital camera has been used intra-operatively through the eye piece of the microscope to record ophthalmic surgery.[3] Herein, I describe a technique of using a regular hand-held video camera for photographic and video documentation of posterior fundus lesions that would be particularly useful to document lesions in infants and children when examined under general anesthesia. Cost-effectiveness of this technique would allow ophthalmologists to acquire reasonable-quality fundus photographs and videos even if they do not have access to a regular fundus camera.

A regular hand-held video camera with a flash mount may be used; higher resolution cameras will offer clearer pictures and videos. A thin torchlight with a bright focused light beam (pen torch) is used as the light source for illuminating the fundus. A home gas lighter holder made of plastic can be modified to slide in to the flash mount of the camera and the torch light is clipped on to the gas lighter holder [Figs. 1 and 2]. Those with a do-it-yourself drive can “sand” the plastic gas lighter holder using a sand paper to enable it to slide easily into the flash holder. This plastic holder will securely hold the torchlight. Alternatively, the torchlight can be secured to the hand-held video camera using stout rubber bands as well, the aim being that the torchlight is as close as possible to the objective of the hand-held video camera lens.

**Figure 1:**
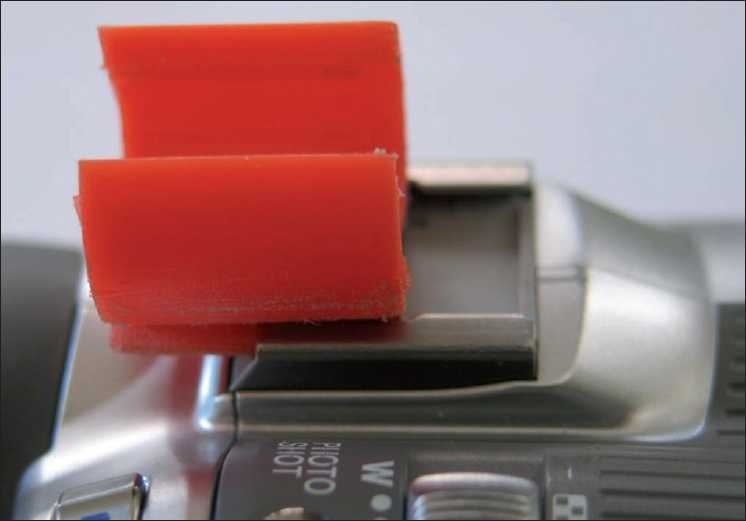
Modified gas lighter holder slid in to the horse-shoe camera flash adapter of the hand-held video camera

**Figure 2:**
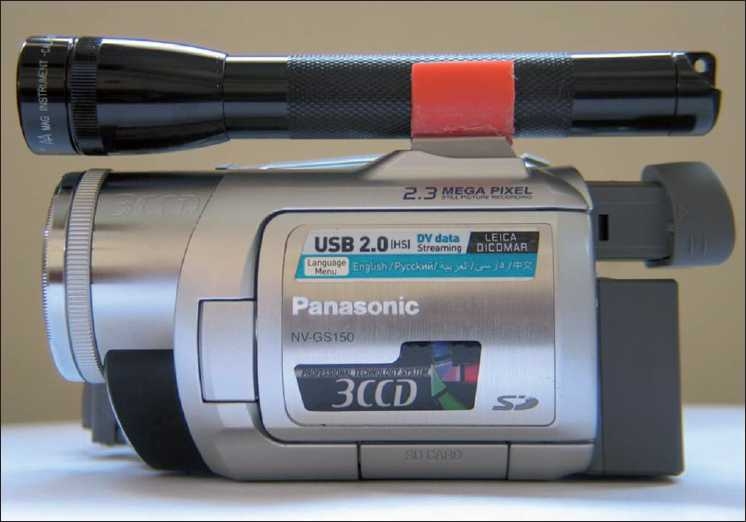
Hand-held video camera with the torch light as illumination source attached to it

## Technique

The technique used to image the fundus is similar to that of uniocular indirect ophthalmoscopy. The torchlight is turned on and focused on a wall at an arm’s length as one would focus the indirect ophthalmoscope – the beam illuminating the upper ½ of the liquid crystal display (LCD) screen.

**Figure 3:**
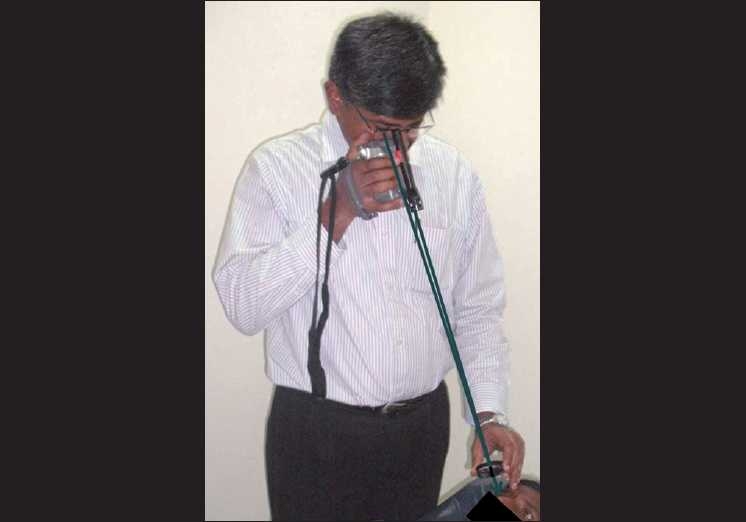
The technique of using the hand-held video camera for fundus imaging

**Figure 4:**
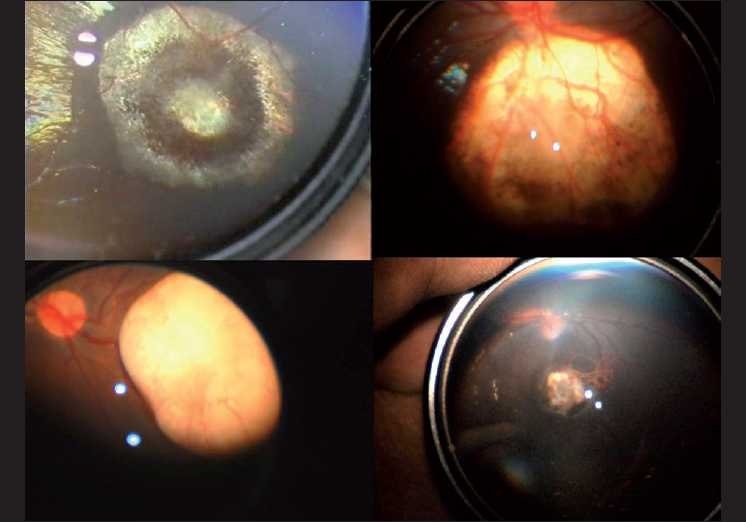
Fundus photographs of pre- and posttreatment retinoblastoma, obtained using the hand-held video camera fundus imaging system

**Figure 5:**
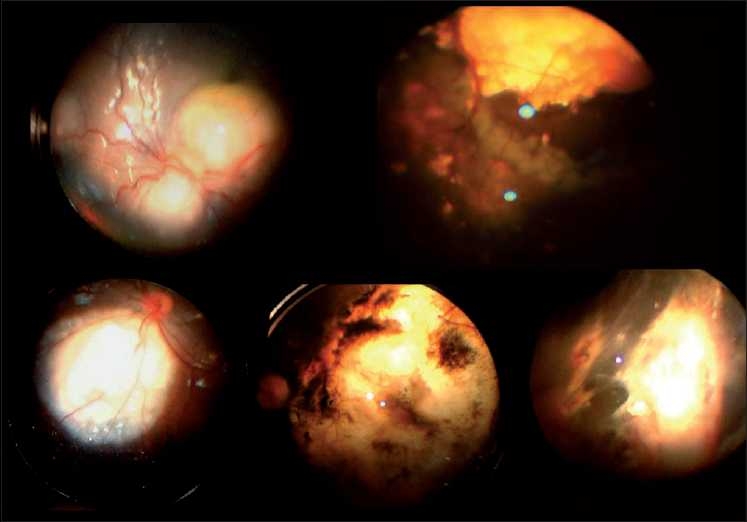
Fundus photographs of pre- and posttreatment retinoblastoma, obtained using the hand-held video camera fundus imaging system

The infant to be examined is administered general anesthesia after pupillary dilatation; if an adult, he / she is made to sit/lie comfortably on the examination chair/couch after pupillary dilatation. Indirect ophthalmoscopy is performed as one would perform a binocular indirect ophthalmoscopy using a condensing lens held in the nondominant hand. The dominant hand is used to hold the hand-held video camera and, using the viewfinder of the hand-held video camera, the external eye is visualized by taking the condensing lens close to the eye [Fig. 3]. The condensing lens is pulled back slowly to image the fundus. Video recording or still photos (if the hand-held video camera offers this facility) of the fundus may be taken by manipulating the hand-held video camera controls. Images of fundus lesions obtained by this technique are illustrated in Figs. 4 and 5.

## Discussion

In contrast to existing fundus imaging techniques, this technique makes use of equipment that is likely to be available with most ophthalmologists and does not require purchase of infrequently used equipment, such as the video indirect ophthalmoscope or the expensive Retcam™. Posterior pole photographs and video recording can be obtained in patients with clear media.

### Limitations of the technique

As in indirect ophthalmoscopy, the light source can create reflexes on the anterior and posterior surfaces of the condensing lens. This can be negated to some extent by minimal tilting of the condensing lens. White balance of the hand-held video camera would also need adjustment to avoid overexposure when capturing an object that is focally illuminated against a dark background – the situation that exists during ophthalmoscopy. The alignment of the torchlight is critical and some hand-held video camera wherein the flash mount is distant from the objective lens may not be suitable for employing this technique. The torchlight should be as close as possible to the objective lens of the hand-held video camera. The auto-flash facility of the camera should be turned off when taking still photos of the fundus.

There is a learning curve when one adjusts to performing uniocular indirect ophthalmoscopy, manipulating the hand-held video camera and the camera controls with one hand and the condensing lens with the other. Aligning the hand-held video camera and the condensing lens to the pupil will need some practice. Using the view finder of the hand-held video camera rather than the LCD screen would help achieve alignment faster as the eyepiece is in line with the objective while the LCD screen is offset from the line of the objective lens. Wearing presbyopic correction aids using the viewfinder to image the fundus. Scleral depression is not possible given that both hands are employed. Peripheral fundus examination is also difficult with troublesome reflexes from the lens and the limitations in focusing of the hand-held video camera.

However, the advantages are least capital expenditure, capability to document fundus images of infants and children under general anesthesia and also the capability to perform a dynamic video indirect ophthalmoscopy in adults.
